# Decolonising the Present by Colonising the Past? A Case From the History of Physical Anthropology

**DOI:** 10.1093/shm/hkae013

**Published:** 2024-07-12

**Authors:** Clemet Askheim, Eivind Engebretsen, Ivar Prydz Gladhaug

**Affiliations:** Centre for Sustainable Healthcare Education, Faculty of Medicine, University of Oslo, Oslo, Norway; Centre for Sustainable Healthcare Education, Faculty of Medicine, University of Oslo, Oslo, Norway; Faculty of Medicine, University of Oslo, Oslo, Norway; Department of Hepato-Pancreato-Biliary Surgery, Oslo University Hospital, Oslo, Norway

**Keywords:** physical anthropology, human remains, decolonisation, race and racism

## Abstract

Inspired by the current transdisciplinary debate about decolonisation, this article raises the fundamental question of how medicine can manage its own history in a way that safeguards the drive for decolonisation, but without eradicating the traces of previous misconceptions. We will do so by reconsidering a case from the complex records of physical anthropology, more specifically, a selected corpus of texts written by the two Norwegian physicians Kristian Emil Schreiner and Alette Schreiner in the early twentieth century, and their relation to race and racism. By teasing out the conceptual nuances and specificities in these texts, we do not attempt to exonerate the Schreiner couple of accusations of racism. Rather, we argue that it is essential to approach the past with caution, avoiding oversimplification when striving to distance ourselves from past thinking

Exploring the management of our own historical narratives lies at the heart of the ongoing debates surrounding decolonisation in various fields, including medicine and medical anthropology. The tragic death of George Floyd, an African American man, at the hands of the police in Minneapolis on 25 May 2020, ignited a wave of protests that led to the toppling, defacement, or vandalism of statues depicting slave traders, generals, and other figures associated with racism and colonialism. In the words of the New York Times, this rejuvenated anti-racist movement has prompted a collective re-evaluation of the past ‘one statue at a time’^1^. By actively rejecting past actions and ideologies, both scholars and the general public are participating in the process of decolonising historical discourses^2^.

Both within the subfield of biological anthropology and anthropology as a whole, there exists a deliberate effort to critically examine the discipline’s historical ties to scientific racism and the adverse consequences it has had on diversity within the field.^3^ A blog post titled ‘Anthropology’s Problematic Past’ succinctly captures this sentiment, stating, ‘Physical anthropology was built on the false concept of the biological authenticity of race and its apparent typological essentialist reality’^4^. While it is unquestionably true that most representatives within the discipline subscribed to these flawed beliefs, it is vital to consider whether this characterisation accurately represents the entire movement. Thus, it is essential to approach the past with caution, avoiding oversimplification when striving to distance ourselves from past thinking. A pivotal question emerges: how can we reassess the past without distorting it or, as Raymond Betts phrased it, ‘was the past, however odious, to be obscured or openly confronted’^5^?

The colonial and racist roots of modern medicine are amongst the historical discourses that have been criticised for many years^6^, and monuments such as the statue of J. Marion Sims, ‘the father of gynecology’, in Central Park, New York, have been removed due to links with slavery and racism^7^. One of the most discredited aspects of the history of the medical sciences is the discipline called ‘physical anthropology’; many of its practitioners became proponents of eugenics and racial hygienic interventions^8^. In many countries, these views resulted in prohibitions on marriage, institutionalisation and forced sterilisations, in the name of purifying hereditary qualities^9^.

Is there a danger of succumbing to similar ideals of purity when we reconsider the past? How can we manage our history in a way that safeguards the drive for decolonisation, but without eradicating the traces of previous misconceptions? These principal questions are the underlying concern of this paper, but rather than engaging directly with current debates, we attempt to shed some new light on the issue by *reconsidering* a case from the complex records of physical anthropology. More precisely, we will analyse a selected corpus of texts written by the two Norwegian physicians and anthropologists Kristian Emil Schreiner (hereafter KE Schreiner) and Alette Schreiner (hereafter A Schreiner) (Figure 1), and scrutinise their use of the concept of race and the meaning of racial differences. The texts selected for close analysis are essays and commentaries written for a broader audience, that reflect the authors’ fundamental thinking about their scientific practice and the relation between their own ideas and those of some other prominent Norwegian contemporary scientists and intellectuals^10^. Moreover, the aim of our analysis is not to use the texts as sources for creating new knowledge about anthropological practices in the period, as this has already been done to a great extent by other scholars^11^, but rather to tease out the arguments and understandings that underpin these practices through hermeneutical analysis of texts. By doing so, we want to challenge our preunderstanding by making an honest attempt to grasp the rationale behind a scientific practice that was prevalent and accepted at the time but has later been rightfully dismissed as unethical and disgraceful. By teasing out the conceptual nuances and specificities in these texts we do not attempt to exonerate the Schreiner couple of accusations of racism. Our aim is not to engage in a debate over whether the Schreiners were, by modern definitions, racists. We unreservedly acknowledge that certain aspects of their anthropological work, especially their measurements of members of the Sami population (an indigenous population living mostly in Northern Norway) in Tysfjord from 1914 to 1921, are profoundly unsettling when assessed through the prism of today’s ethical norms. Yet, it remains vital to explore the intricacies and nuances within the Schreiners’ positions. Though their practices and beliefs may not only strike a modern reader as problematic, but indeed deeply unsettling or offensive, we contend that their perspective was not grounded in the ‘concept of the biological authenticity of race and its apparent typological essentialist reality’. In lieu of reducing their viewpoints to a monolithic label of racism, we aim to delve deeper into their contentions, striving to present a more comprehensive and nuanced understanding.

**Fig. 1.**
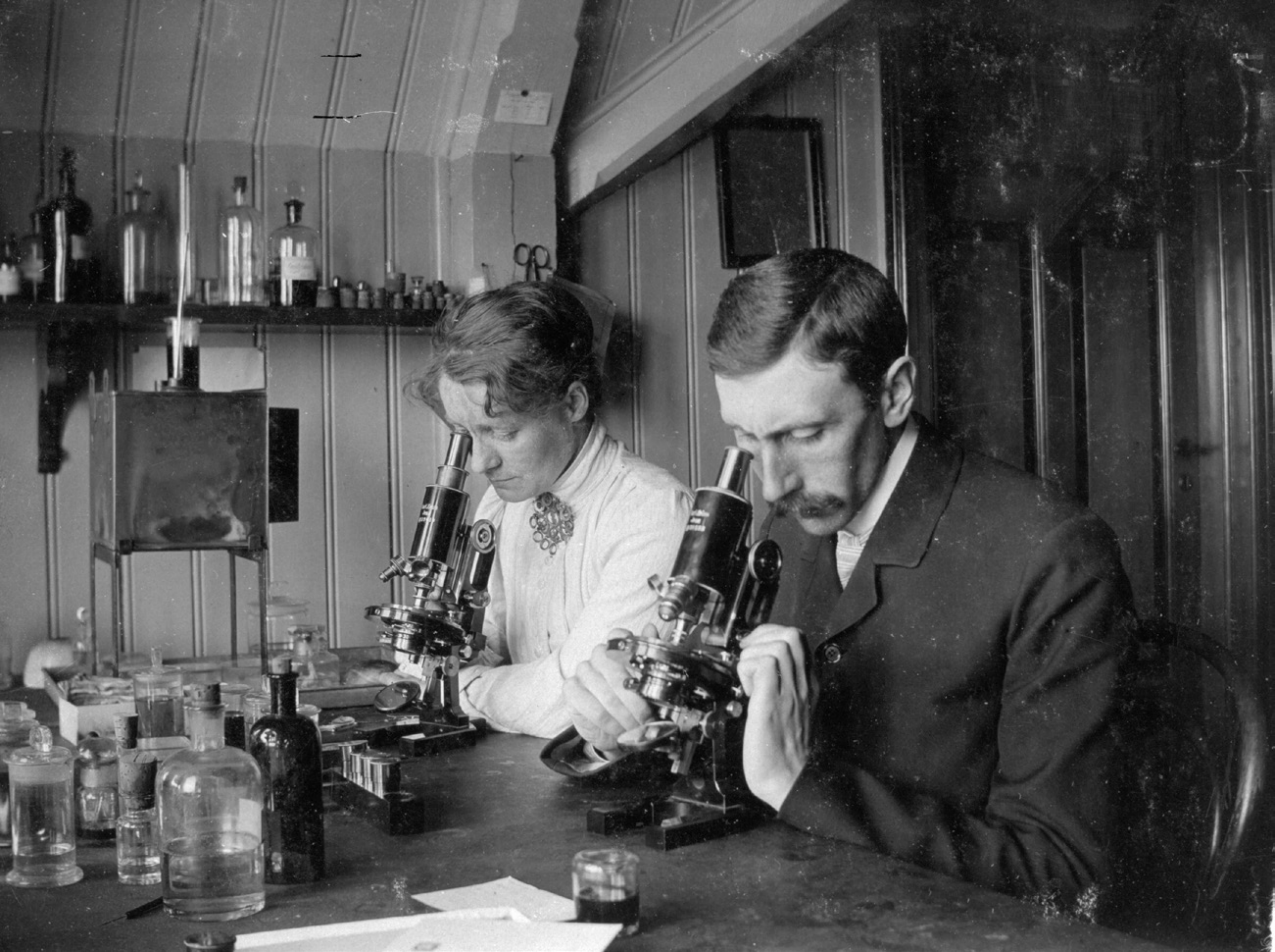
Alette Schreiner (1873–1951) and Kristian Emil Schreiner (1874–1957) at their laboratory bench, Drøbak Biological Laboratory, 1906. source: The Museum of University History, Museum of Cultural History, University of Oslo.

The work of professor KE Schreiner and his wife A Schreiner was seminal to the development of physical anthropology in Norway and Scandinavia. They both published widely on this topic in several languages and together they contributed extensively to a large collection of human remains (the Schreiner collection) that still exists. Recently, this collection, as similar collections worldwide, has become the object of both academic and popular criticism, linking their contributors to accusation of racism in a modern sense.^12^ The case of KE and A Schreiner, and physical anthropology more generally, is emblematic for the question of how to manage history since the thoughts and practices the case describes are so alien and badly connoted to modern readers. Despite—or rather because of this estrangement—we believe that it is important to study their concepts and arguments in order to discern their underlying presuppositions.

## Collection of Human Remains

In the mid-ninteenth century, European and American scientists started to collect human remains in order to study morphological differences and explore human biological history. The scientific motives for exploring issues of human origin, racial comparisons and evolution, were from the beginning intertwined with more political motives, such as the justification and legitimation of colonialism and imperialism.^13^ If the native populations in the colonies were truly inferior savages, then ‘the white man’s burden’ could be seen as a virtuous undertaking: ‘You do not have to treat people equally, if they are sufficiently different’.^14^

Many of the collected remains came from different indigenous groups or were of non-European origin,^15^ and the collectors would ‘step over corpses’ to get hold of rare specimen that could enrich their collections.^16^ In 1988, the American Association of Museums reported to hold 43,306 Native American skeletal remains: ‘The Smithsonian Institution alone had 18,600 American Indian and Alaskan native remains’.^17^ Many of these are today repatriated^18^ and the nineteenth-century collecting practices have been dismissed as disrespectful.^19^ In 1990, the Native American Graves Protection and Repatriation Act (NAGPRA) was passed, stepping up the pace of repatriations.^20^ In 2020, the US National Park Service estimated that during its 30 years of existence, this legislation had facilitated the return of about 67,000 human remains, but also that the remains of some 127,000 Native Americans were still in the possession of American museums.^21^

Repatriation has nevertheless remained controversial due to the potential loss it entails for research opportunities within fields such as disease history, migration, osteology, forensic science and human archeology.^22^ ‘The effects of repatriation requests and decisions, especially on North American collections, will unquestionably lead to loss of skeletal collections’.^23^ Ubelaker and Grant even claim that ‘much of what is known today about Indian history has been learned through the study of human remains’.^24^ There have also been conflicts between museological interests and demands for repatriation.^25^ Concerns about museological interests and loss of research opportunities have also been raised in Norway, related to the Schreiner collection.^26^

The most well-known case to date was the reburial in 2011 of skeletal remains from an excavation in Neiden in Northern Norway^27^, where in 1915, despite local protests, remains from 94 Sami individuals were unearthed and brought to the Institute of Anatomy at the University of Oslo.^28^ However, the repatriation and reburial process was time consuming, and also controversial even in the local Sami community. Most of the local Sami inhabitants seemed to be satisfied and relieved, while some lamented the potential loss of scientific knowledge caused by the reburial^29^: ‘Once they have come out of the ground we are obliged to do research on them so that the excavation has not been in vain’.^30^ Some also questioned whether this was the right way to manage their history: ‘Now we are burying our own history’.^31^ Or in the words of Sami politician Ole Mathis Hætta:

The injustice which occurred when they [the skeletons] were collected will not be healed when, many years later, we do something unwise. On the contrary, being preserved in museums (not necessarily as an exhibition) they will be a testimony to future generations about what happened previously. By burying them we are participants in falsifying and embellishing history.^32^

## Physical Anthropology in Scandinavia and Norway

Researchers from Scandinavia in general played an active part in the growing research on race, heredity and racial differences throughout the rise of physical anthropology and its later development into eugenics and racial hygiene. The Swede Carl Linnaeus (1707–1778) divided the human species into four different races (or varieties) in the great taxonomic system he introduced in *Systema Naturae* in 1735.^33^ In the present standard historical narrative of scientific racism, the inclusion of man in this taxonomy has been considered a fateful event.^34^ His countryman Anders Adolf Retzius (1796–1860) introduced the cephalic index (a cranial index based on the ratio between skull breadth and width) in 1840,^35^ paving the way for racial classifications based on skull measurements. He divided humans into brachycephalic and dolichocephalic types, later adding an intermediary type, mesocephaler.^36^ In Denmark, the famous biologist Wilhelm Johannsen (1857–1927) was crucial in the development of early genetics, inventing the terms ‘gene’, ‘genotype’ and phenotype’.^37^ And in Norway the biologist Kristine Bonnevie (1872–1948) was a highly regarded researcher in genetics and made significant contributions to the study of papillary patterns (fingerprints) and inheritance.^38^

Several academic institutions in Denmark and Sweden host big collections of human remains (e.g. University of Copenhagen,^39^ University of Uppsala,^40^ University of Lund^41^ and Karolinska Institutet^42^), but the largest and most comprehensive in Scandinavia is the one named after KE Schreiner and located at the Medical Faculty at the University of Oslo.^43^ A part of the collection contains remains of Sami origin, which since 2020 is administrated by the Sami Parliament. In total, the collection numbers more than 8,000 remains, of which about a thousand are of Sami origin^44^. So far 97 of these have been repatriated upon request from the Sami Parliament, of these 94 from Neiden as explained above^45^ and 3 additional upon separate requests.

Both KE Schreiner and A Schreiner studied medicine at the University of Oslo and their first collaborative research project was on cell division and germ cells. In 1908, KE Schreiner was appointed professor of anatomy and Director of the Institute of Anatomy at the University of Oslo. The following years he developed an increasing interest in physical anthropology, heredity and racial differences, and as a result, he started to collect human remains.^46^ In 1914, A Schreiner published a book on heredity in humans,^47^ and during the following decades both she and her husband were engaged in a huge anthropological study of presumed racial differences among rural populations across Norway. They measured heads and height, noted hair and eye colour, and other racial traits. The results were published in 1929 under the title *Die Somatologie der Norweger*, together with their collaborator at the time, the Norwegian army physician Halfdan Bryn (1864–1933).^48^

Later they initiated a study of the physical anthropology of the Sami people in Northern Norway, published in 1935 as *Zur Osteologie der Lappen.*^49^ For this study, they collected hundreds of remains from Sami people in Northern Norway, where most of the remains were dug up from different graveyards and burial sites chiefly in the northernmost county of Finnmark.^50^ The most well-known example is the excavations in Neiden mentioned above. These were approved and supported by the government and commissioned by KE Schreiner and took place in the summer of 1915.^51^ This practice of grave desecration was widespread within physical anthropology at the time, and has later been condemned.^52^ As pointed out by Highet, ‘[o]ver the last century, standards have been developed and boundaries established to ensure that blatant violations of the sanctity of the grave are not permitted simply to amass raw data for study’.^53^

The Sami people, or Lapps as they used to be called, had ever since Linneaus been a group of special interest to physical anthropologists. Their origin had remained a mystery, and likewise how they should be racially classified.^54^ Indigenous people were generally of special interest to physical anthropologists, since these people were believed to be racially purer than ‘civilised’ and more bastardised populations.^55^ This view was partly based on the idea that ‘[d]ifferent races belonged to different natural environments’^56^, and that racial mixing and modern lifestyles weakened the symbiotic relationship between man and nature.

Besides the cultural and historical questions of origin and heritage that had been debated by historians, linguists and anthropologists for years^57^, the question of indigenousness also had political implications. How to deal with the Sami population was a recurring issue in both Norway and Sweden and different scientists and disciplines had diverging opinions at various points in time, regarding their status.^58^ One curious example is the debate between the Norwegian and Swedish governments, following the dissolution of the Norwegian-Swedish union in 1905, about the rights of Swedish Sami nomads to move their reindeer herds to summer pastures on Norwegian territory. The Swedish government argued that the ‘Lapps’ were an indigenous people who should be allowed to uphold their traditional lifestyle, whereas the Norwegian government denied the aboriginal status of the Sami, arguing that they should abandon their backward culture and not hamper an expansion of Norwegian agriculture.^59^

The Schreiner’s were physicians and worked from within the ruling paradigm of physical anthropology at the time, summed up in Rudolf Martin’s *Lehrbuch* from 1914.^60^ Here anthropology was commonly understood as ‘the natural history of man’^61^, equated by Martin as ‘the study of race’^62^. ‘[Y]et race for Martin, unlike subsequent anthropologists, was a purely physical concept’, based on morphological differences and divided into racial types^63^.

In their study of the Sami in Tysfjord in Northern Norway, the Schreiners seem to have been looking for such a racial type, but without success^64^. Instead of concluding that racial types do not exist, they displaced the idea of the pure racial type further back in the evolutionary prehistory, thus concluding that the type was bastardised and mixed with elements from other racial types. This shows that they took their empirical results, or rather lack thereof, seriously enough to change their theory, yet largely remaining within the paradigm.

## Hierarchy and the Meaning of Primitive

Stephen Jay Gould distinguishes between *anthropology* as a scientific tradition concerned with racial classifications based on physical measurements, *racial theories* placing the different races in a hierarchy, *scientific racism* as using measurements from anthropology to support racism, and *racial discrimination* which refers to the practical consequences of racism, including scientific racism.^65^ If we consider some of the works by KE and A Schreiner from the early twentieth century with Gould’s analytical categories, it seems obvious that their classifications and taxonomies imply hierarchies of traits, differences and characteristics. This is in line with the contemporary concept of physical anthropology as a science of race. However, the question is what kind of meaning and status the Schreiners attributed to these hierarchies. Some authors writing about the history of physical anthropology seem to take for granted that when racial differences were the object of study, it was always in relation to a hierarchy of races, according to which some races were considered inherently better than others.^66^ Such a tendency to equate everything racial with racism has been prevalent in recent public debate in Norway^67^.

It seems difficult to argue against the Schreiners being racists, since words like ‘primitive’ and ‘infantile’ were frequently used in anthropological literature^68^, also by A Schreiner: ‘As usual with primitive peoples, the Lapps, in spite of their childlike curiosity and friendliness, were downright unwilling to subject themselves to a detailed examination, especially if this involved undressing’.^69^ And also by KE Schreiner: ‘The carelessness often encountered among the Lapps, sometimes with a childish confidence, sometimes with great shyness, and not quite seldom with a complete irresponsibility, agrees with the somatic type, and directs the thoughts towards the protomorphic races of Eurasia’.^70^

However, such concepts are not necessarily a *judgement* of mental abilities. In a study of the Danish anthropologist Søren Hansen (1857–1946), the historian Poul Duedahl comments on the use of ‘primitive’: ‘The concept of primitive did for Søren Hansen (as for many of his contemporaries) have a neutral meaning, but it relied, as with Paul Broca, on the predisposition that the white race was the norm that the other races were measured against[…]’.^71^ In the history of ideas, characteristics such as infantile and primitive are frequently used to describe stages of civilisation, without necessarily implying a general normative judgement. For instance, in Christian tradition, we find the idea of six historical epochs and their analogy with six individual ages: infancy, childhood, youth, early manhood, later manhood and old age.^72^ In his famous essay on cannibals, the renaissance humanist Montaigne idealises the natives of ‘the new world’, comparing their alleged purity with the barbarian practices of torture in Europe.^73^ And in his reaction to the estrangement of the civilised and cultivated Europeans, Rousseau romanticises people living in a ‘state of nature’.^74^ These are all examples showing that analogies to primitive and infantile stages of development are not necessarily understood in purely negative terms.

The use of infantile, young, incomplete, primitive, etc. is also very much in line with the evolutionary vocabulary which was widely used at the time.^75^ As discussed below, KE Schreiner even described racial hygiene in evolutionistic terms, as a ‘young and incomplete’ science.

## Evolution and Race

Reinhart Koselleck has famously described modernity ‘as a shift from one experience of time and history to another’ through which ‘space of experience’ [Erfahrungsraum] was gradually distinguished from ‘horizon of expectation’ [Erwartungshorizont].^76^ More specifically, this implies that knowledge of the past could no longer be used as a basis for predicting the future, or knowing what to expect, which in turn led to an almost obsessive interest in the future in terms of anticipations, plans, and prognoses.^77^ This modern sense of temporality received a clear formulation in the philosophy of history developed by Kant, Herder and others at the end of the eighteenth century.^78^ According to Koselleck, this general intellectual shift led to a redefinition of several basic concepts, such as history, and related scientific practices, such as historiography, in which the emphasis on the collection and categorisation of past experiences was replaced by a new emphasis on evolution, progress and predictions of the future.^79^ Subsequently, the concept of race was gradually incorporated into the new ideas of progress and evolution, where the focus on merely describing and categorising racial traits was gradually replaced by ideas of anticipating and planning racial evolution.

Aided by the philosophy of Herbert Spencer and Auguste Comte, the idea of evolution left the confines of biology and became a generalised ideology of evolutionism.^80^ In this period, cultural and psychological differences between peoples became welded onto the concept of race, assuming a correspondence between outer and inner traits.^81^ Such a correspondence was not only the starting point for phrenology, but a common assumption among racial scientists, making normative racial hierarchies even more compelling.

This assumption is reflected in the existing literature on the Schreiners and their scientific work^82^. As Evjen points out: ‘Obviously, the researchers [the Schreiners] were influenced by current thinking on racial hierarchy’^83^. Schanche is even more explicit in her comment on KE Schreiner’s ‘opposition to the emerging Nazi race ideology of his day’: ‘However the thesis of the superiority of the Nordic race was implicit in the schemata within which he [KE Schreiner] expressed himself. He was part of a research tradition which could only produce outcomes that we today cannot characterise as anything but racist’.^84^ Nevertheless, in the texts we have analysed, race and culture are separated, and both racial hierarchy and the superiority of the Nordic race are explicitly denied. To be more precise, the Schreiners reject the notion of a racial hierarchy that is perceived as an ‘inherent typological essentialist reality’. Insofar as the Schreiners may entertain the concept of a racial hierarchy, it is not conceptualised as a ranking of disparate population types. Instead, as will be further expounded upon below, it is perceived within the framework of the intricate dynamics and reciprocal influences of various traits disseminated across a multitude of population groups.

It is important to underline that after Darwin, and especially after the rediscovery of Mendel, the biological underpinnings of the concept of race were weakened.^85^ There is no room in evolution for race as an object of analysis. There are species and there are inherited traits, but no race in purely biological terms. It can only be a meaningful biological category if it is seen in terms of adaptation to specific environments. In this sense ‘every race was at the top of the hierarchy in the geographic area where it resided’.^86^ Thus, there are hierarchies, but local ones, indicating that some races are better adapted to the environment in which they live, as also indicated by Evjen in the earlier quote about different races belonging to different environments.^87^ Ranking natural environments is meaningless, and individual organisms or races are only comparable, as to how well they are adapted to the environment in which they live. The concept of a hierarchy among races is dependent on the influence of culture, psychology and ‘civilisation’. In the case of Schreiner, a distinction was made between the concept of race and the concepts of people and culture:

It must be pointed out immediately that just as there is no Norwegian or English race, there is no Jewish race. The Jews, like Norwegians and Englishmen, are a people, composed of several races, but bound together by common traditions, customs and most of all a common religion.^88^

In a radio lecture from 1932, KE Schreiner commented on the relation between ‘outer’ and ‘inner’ traits as follows: ‘As I now will end with a few words about the racial characteristics of the soul, I must immediately point out that what confronts us here are questions that are far from scientifically clarified and which are partly treated very subjectively in the literature’.^89^ He obviously considered these issues as speculations rather than real science. The main objective of Schreiner’s scientific endeavour was to systematise and classify *physical* traits. To connect such traits with inner qualities and values, and to place them in a normative hierarchy, was not only outside the scope of his research but a practice that he explicitly described as unscientific:

The prominent role played by Europe in the history of cultural development is not reducible to a single race but has rather come about through a successful interplay of races, reciprocally complementing and stimulating each other. To glorify one race to the detriment of the other must therefore be termed unscientific. And when talking about scientific racial hygiene, one should bear clearly in mind that the word race should not be associated with any specific anthropological race, but rather with the more or less positive and negative hereditary traits contained in all races, and of course to an even larger degree, in all peoples.^90^

In line with contemporary development within evolutionary theory, Schreiner emphasises that cultural superiority cannot be attributed to one ‘anthropological race’. Nor is superiority linked to some kind of biological essence but to *cultural* development. Schreiner associates this development with a hierarchy of traits, in which some traits become ‘more positive’ than others. While using words such as infantile, primitive, etc. to classify human cultures, Schreiner also states that races are not to be confused with ‘anthropological races’, but a mixture of more or less superior traits existing in all races, and that ‘to glorify one race to the detriment of others is unscientific’.^91^

Instead there seems to be two different concepts of culture at work in the writings of the Schreiners, denoting different objects. Inspired by the Danish historian of ideas Johannes Sløk, we could name them *culture as performance* and *culture as self-expression*.^92^ While culture as performance refers to cultural production in terms of technologies, art, literature and so on, culture as self-expression denotes ways of living and expressing oneself (e.g. lifestyle). Such a distinction would make it possible for the Schreiners to refer to a hierarchy on the level of cultural *performance* through expressions such as ‘infantile’ or ‘primitive’, while at the same time explicitly denying the presence of general hierarchies of *life expressions*. This would amount to a form of Eurocentrism, but not necessarily racism, as defined by Gould (above).

## Eugenics

In the context of a generalised evolutionism, the field of eugenics was born, in close relation to the discipline of physical anthropology. To navigate through this period we must distinguish between three different analytical objects, as they were perceived at the time: the established scientific discipline of physical anthropology, the potential science and contested research field of eugenics, and eugenics or racial hygiene as a political project.^93^

The term eugenics was coined by the statistician Francis Galton (1822–1911) in 1883, as a ‘brief word to express the science of improving stock’,^94^ and it was quickly picked up by political reformers.^95^ The basic idea was that modern industrial society had thwarted natural selection, as Charles Darwin had described it, and that inferior individuals survived and reproduced to an extent unfavourable to society at large. The political task was therefore to reverse this degenerative development by means of positive, negative and prophylactic, racial hygiene.^96^ The reduction in birth rates in many European countries since the turn of the century made the task seem urgent, for what if the wrong people reproduced the most? In Norway, and in Scandinavia in general, this project appealed to progressives and radical reformers,^97^ who wanted to ‘improve the biological quality of the population in the interest of the state and of future generations’.^98^ However, this project contained several internal contradictions, which we also find traces of in the evolving views and attitudes of the Schreiners.

The Schreiner couple were first and foremost scientists, and of a positivist and evolutionist inclination, and they were primarily concerned with describing, measuring, comparing and classifying external features of the human body. Their research activity within physical anthropology, as well as their attitudes towards both research and racial differences, can be seen as exemplary for physical anthropology in this period. Sharing some of the basic ideas of racial hygiene and eugenics, they later opposed the practical and political proposals made in its name, because they believed it lacked sufficient scientific backing. The political implications of this ‘modern science of the culture of the human race’, as A Schreiner called it in her book on the genealogy of humans from 1914,^99^ were yet to be fully realised. Nevertheless, she devoted a whole chapter to racial hygiene in the book, in which she argued in favour of the basic thrust of eugenics, as ‘a replacement for “natural selection”’ in human societies where the cultural development has ensured the survival of weak individuals who would not survive under natural conditions.^100^ On a more concrete level, she endorsed negative eugenic measures such as sterilisation and abortions, although without actively promoting such practices.^101^ At the same time she underscored the importance of nurture and the material, social and cultural conditions in which individuals are brought up.^102^ We must, however, note that history is a temporal affair and that time passed, also in the past. Accordingly, the Schreiners’ position on eugenics was not a static or fixed matter, but changed over time.

At this point (around 1915), her husband seems to be more reluctant than her towards discussing potential political implications of racial hygiene: ‘As an exact science, racial hygiene is still very young and incomplete. It is still at the stage of just being a collection of empirical data and is thus unsuitable as a foundation for practical politics. Dilettantes, however, have no regard for this difference’.^103^ He was concerned by the scientific infantilism of the eugenic project and criticised the hubris that some of his research colleagues expressed on its behalf. As such, he was an early sceptic, but he was soon joined in his scepticism by both his wife and the majority of the academic community in Norway, including Kristine Bonnevie.^104^ As scientists they acknowledged the new research on genetics and heredity that took place during the inter-war years,^105^ in contrast to the more ideological proponents in the eugenic movement,^106^ which led to a schism in the scientific community in Norway and in Scandinavia in general.^107^ The rise of national socialism in Germany, including their views on racial science, also contributed to the falling scientific credibility of eugenics.^108^

## No Nordic Master Race

As both KE and A Schreiner felt a deep obligation to their scientific ideals, they became increasingly critical of their former partner Dr. Bryn, who they felt drew too far-reaching racial conclusions about heredity based on scant evidence. They also criticised other racially oriented eugenicists for divergence from what they believed to be the proper conduct of science.^109^ They did not share Bryn’s concern for the purity of the Nordic race, nor did they share his views on eugenics. Bryn belonged to a group that advocated radical eugenic measures, promoting the superiority of the Nordic race and according to KE Schreiner, practicing pseudoscience.^110^ The most active spokesman for these views in Norway was the pharmacist Jon Alfred Mjøen (1860–1939), who held a prominent position in the international eugenic movement and was one of the founders of The International Eugenics Organization in 1912.^111^ Both Bryn and Mjøen had ties to more radical eugenicists such as Eugen Fischer and Hans Günther in Germany, Charles Davenport, and Madison Grant in the US, and Herman Lundborg in Sweden.^112^

Schreiner connected races with traits or elements rather than with people and culture, as Bryn and other more nationalistically oriented researchers had done. Such traits are traceable across various population groups:

That people with an ancient culture, such as the Sumerians, Babylonians, Assyrians, Egyptians, Greeks, Romans and Arabs should contain Nordic racial elements is partly more or less likely, partly certain; that their cultural development depended on these elements, are however quite dubious.^113^

In this quote, Schreiner emphasises that the Nordic race is far from unified but consists of ‘elements’ that are present across several populations that are often taken to be very different. At the same time, he explicitly refutes the idea that the cultural development among these groups can be traced back to these traits. In other words, the Nordic race is neither unified nor superior.

After the publication and dissemination of Thomas Hunt Morgan’s chromosome theory and Johannsen’s work on genotype and genes, a more holistic approach to genetics and heredity was called for.^114^ Ideas of racial purity and superior pure races became increasingly questionable from a scientific point of view. During their study on the racial characteristics of Norwegians, KE and A Schreiner came to believe that races were just a typical mixture of traits and adaptations. According to Kyllingstad, KE Schreiner claimed that the term ‘Nordic race’ ‘was a descriptive term designating a “phenotype” that had occurred in northern Europe as a product of racial mixing during and after the Neolithic’.^115^ Instead of retaining previous views of races and heredity, and adjusting the data accordingly, as many others had previously done,^116^ KE and A Schreiner instead seemed to have adjusted their views according to new theories and new data.

## Self-Expression or Performance?

Counter to the evolutionary dogmatism of Bryn and other promoters of the Nordic idea, A Schreiner toned down self-preservation and struggle for survival as the essential evolutionary forces. Instead she proposed a vitalist theory of evolution according to which there is a drive towards self-realisation in all biological development.^117^ If an environment becomes too harsh or the struggle for life too hard, selection might produce species that are too specialised, and thus incapable of adopting to changing environments. One of her examples of such evolutionary dead ends are the dinosaurs, which she compares to the Nordic race:

Maybe it is the Nordic race—the master race of the blue Viking and of warrior blood, praised by poets and others as the most glorious product of the struggle against a harsh environment and the proof of this struggle as the only fruitful means of development—who will be the next to run our head against the wall!.^118^

Her critique of certain forms of evolutionary thinking developed into a comprehensive and radical philosophy of life that in many ways echoed contemporary vitalist ideas.^119^

In 1933, the biologically oriented Norwegian philosopher Peter Wessel Zappfe wrote an essay titled ‘The Last Messiah’, where he made similar statements, only his object was the human species as a whole and not individual races:

Whatever happened? A breach in the very unity of life, a biological paradox, an abomination, an absurdity, an exaggeration of disastrous nature. Life had overshot its target, blowing itself apart. A species had been armed too heavily—by spirit made almighty without, but equally a menace to its own well-being. Its weapon was like a sword without hilt or plate, a two-edged blade cleaving everything; but he who is to wield it must grasp the blade and turn the one edge toward himself. ^120^

A Schreiner speculates whether the Nordic race has become to specialised for struggles and hardship, while for Zappfe it is the human species that has become too smart for its own good, loosing its natural innocence and peace of mind, instead becoming alienated and anxious.

According to A Schreiner, ‘expressions of life’ and ‘excess of life’ cannot easily be submitted to a rational order of utility, evolution and survival:

From their utilitarian point of view, the men of knowledge have tried to reduce the beautiful song of the nightingale and the song thrush, and the melody of the lark rejoicing the spring from high above, to an instrument in the service of reproduction, rather than simply accepting these expressions of life, like human song and play and laughter, as spontaneous eruptions of a personal excess of life, as the urge to voice ones inner mood.^121^

She further develops her arguments as follows:

The first expression of life that recklessly spilled it’s powers, was like the first dawning of a child’s smile over the previously solemn Earth. The first breath of life’s free display, which little by little, as the Earth is spinning around the Sun, should expand into the perfect storm we call human life.^122^

This argument resembles later critiques of reductive evolutionary biology; what Gould and Lewontin jokingly called ‘the Panglossian paradigm’ (after the character Pangloss in Voltaire’s *Candide*): ‘the proposition that each part [of an organism] is “for” some specific purpose’.^123^ From this perspective it seems misplaced to try to fit expressions of life such as people’s song, laughter and joy into a hierarchy. Although A Schreiner sometimes seems to suggest the existence of subordinate races^124^, this viewpoint is not consistent throughout her work.

## Physical Anthropology, Racism and Present Interpretations

If one considers physical anthropology in the early twentieth century, there are at least three principle issues that should be subject to criticism:

First, it represented an apparent scientific backing that could be used in racist ideology to legitimate colonialism, imperialism and discrimination. Second, it produced scientific arguments for, and partly promoted, eugenics and racial hygienic interventions and policies that caused considerable suffering for several minorities. Third, it used dubious and highly unethical research methods, both in collecting human remains from burial sites, and in performing various measurements on living human beings who neither understood nor could easily oppose what was being done to them.

Of these problematic issues, at least the third is applicable to KE and A Schreiner, and perhaps the first and second as well, if one looks at their total contribution throughout their careers. But they have equally contributed to discrediting supposedly scientific arguments for eugenics, as in the second case, and also to de-legitimise unequal treatment of people based on conceptions of biological race, as in the first case. Scientifically, KE and A Schreiner succeeded in discrediting and marginalising their more extreme colleagues in physical anthropology. As such, they made racial science in Norway and Scandinavia less racist. However, their positivist scientific ideals of objectivity and neutrality, free from politics and more mundane concerns, did not prevent racial discrimination of the Sami population following in their footsteps. Neither did they argue against the passing of a Norwegian Sterilization Law in 1934^125^, which legitimised sterilisation of mentally retarded, sexual criminals and the insane, based partly on an idea of eradicating undesirable hereditary traits^126^. We cannot suggest that the Schreiners were anti-racists, but it is equally wrong to postulate that they were racists in a modern sense of the word. On the one hand, we could say that the Schreiners’ embeddedness in the research tradition of physical anthropology, following Martin and others, makes their work tainted by that legacy, and therefore racist. This is true irrespective of whether they themselves had an awareness of these issues, or were in agreement with the core postulates on race in the tradition from which they were working. On the other hand, they actually took an active stand, although on a purely scientific basis, against certain racist ideas and eugenic policies. Especially A Schreiner seemed partly to have worked from vitalist ideas fundamentally at odds with hierarchies based on any biological conception of race. The Schreiners were clearly Eurocentric, and as such ‘products of their time’. However, being a product of one’s time is neither an excuse,^127^ nor a fixed determination. Treating all racial science as simply racist, makes it hard to see the nuances and to acknowledge that it was a field of serious research, scientific curiosity and political controversy.^128^

## Conclusion

We maintain that decolonisation strategies should avoid conceptual simplification and anachronisms. If we in our efforts to make amends and moral improvements, instead create a simplified image of the past as the colonial or racist other to be conquered, we risk colonialising the past by taking for granted the supremacy and universality of our own present knowledge, concepts, sentiments and values. Furthermore, such an approach to the ideas of the past tends to treat important concepts, such as race, as static and homogenous meaning units.^129^ Moreover, by distancing us from the past we tend to keep the present not liable to similar misdemeanours, shifting the attention to an ignorant past that we now have moved beyond. In the case of racism we might self-righteously acquit ourselves of any accusations, by believing that it is a surpassed stage: we used to be racists, but now we know better. At worst, we risk making contemporary instances and experiences of racism into exceptions, caused by ignorance, propaganda or immorality. It is only by understanding the past in positive terms and not as a negation of our own ideals and values,^130^ that history can function as a correction and question mark towards our own time, challenging current views and practices.

## Footnotes

1 S. Mervosh, S. Romero, and L. Tompkins L. ‘Reconsidering the Past, One Statue at a Time’, *New York Times*. 16 June 2020. https://www.nytimes.com/2020/06/16/us/protests-statuesreckoning.html?action=click&module=RelatedLinks&pgtype=Article

2 W. Anderson, ‘Decolonizing Histories in Theory and Practice: An Introduction’, in *History and Theory: Studies in the Philosophy of History*, 2020, 59, 3, 369–75; Finn, G. M., Danquah, A. and Matthan, J. ‘Colonization, Cadavers, and Color: Considering Decolonization of Anatomy Curricula’. *Anatomical Record* (Hoboken, NJ: 2007), 2022, 305, 4, 938–51; Eichbaum, Q.G. *et al.* ‘Decolonizing Global Health Education: Rethinking Institutional Partnerships and Approaches’, *Academic Medicine*, 2020, 96, 3, 329–35.

3 Antón, Susan C., et al. ‘Race and Diversity in U.S. Biological Anthropology: A Decade of AAPA Initiatives’. American Journal of Physical Anthropology, 2018, 165, S65, 158–80.

4 University of Toronto, Department of Anthropology, ‘Anthropology’s Problematic Past’ (https://www.anthropology.utoronto.ca/about-us/our-department/anthropology-and-diversity).

5 R. F. Betss, ‘Decolonization: A Brief History of the Word’ in E. Bogaerts and R. Raben, eds, *Beyond Empire and Nation – The Decolonization of African and Asian Societies, 1930–1970s*. (Leiden: Brill, 2013), 35.

6 P. Farmer, P. Drobac, and Z. Agoos, ‘Colonial Roots of Global Healt’, *Harvard College Global Health Review*. 19 September 2009. https://hcghr.wordpress.com/2009/09/19/colonial-roots-of-global-health/; K. Wailoo, ‘Historical Aspects of Race and Medicine: The Case of J. Marion Sims.’ *JAMA: The Journal of the American Medical Association*, 2009, 320, 15, 1529–30.

7 C. Domonoske, ‘Father Of Gynecology, Who Experimented On Slaves, No Longer On Pedestal in NYC’. 17 April 2018. *NPR*https://www.npr.org/sections/thetwo-way/2018/04/17/603163394/-father-of-gynecology-who-experimented-on-slaves-no-longer-on-pedestal-in-nyc?t=1612693678427&t=1613895795862; Wailoo, ‘Historical Aspects of Race and Medicine’.

8 Proctor, R. ‘From Anthropologie to Rassenkunde in the German Anthropological Tradition’, in Stocking, George W, ed, *Bones, Bodies, Behavior: Essays on Biological Anthropology*. *vol*. 5 (Madison, Wis: University of Wisconsin Press, 1988), 138–79; Massin, B. ‘From Virchow to Fischer. Physical Anthropology and “Modern Race Theories” in Wilhelmine Germany’ in Stocking, George W, ed. *Volksgeist as Method and Ethic: Essays on Boasian Ethnography and the German Anthropological Tradition*. *vol*. 8. History of Anthropology (Madison, WI: University of Wisconsin Press, 1996), 79–154.

9 Kevles, D. J. In the Name of Eugenics – Genetics and the Use of Human Heredity (Cambrigde, Mas: Harvard University Press, 1995). Evjen, B. ‘Measuring Heads: Physical Anthropological Research in North Norway’, *Acta Borealia*, 1997, 14, 2, 3–30; Koch, L. ‘Past Futures: On the Conceptual History of Eugenics – A Social Technology of the Past’ in *Technology Analysis & Strategic Management* 2006, 18, 3–4, 329–44; N. Roll-Hansen, N. ‘Eugenics Before World War 2: The Case of Norway.’ *History and Philosophy of the Life Sciences*, 1980, 2, 2, 269–98; N. Roll-Hansen, ‘Norwegian Eugenics: Sterilization as Social Reform’, in Broberg, G and Roll-Hansen, N, eds, *Eugenics and the Welfare State – Sterilization Policy in Denmark, Sweden, Norway, and Finland* (East Lansing: Michigan State University Press, 1996), 151–94.

10 Alette Schreiner, *Slegtslivet hos menneskene* [Heredity in Humans], Kristiania: Aschehoug & co, 1915; KE. Schreiner, ‘Europas menneskeraser [The Races of Europe]’ in *Mennesket som ledd i naturen – Universitetets radioforedrag* [Humans As Part of Nature – Radiolectures From the University], serie A, nr. 5, Oslo: Aschehoug & co, 1932; KE Schreiner, ‘Et ord om videnskap og dilettantisme [A Word on Science and Dilettantism]’, *Norske Intelligenssedler*, 1915, 8 April; A. Schreiner, ‘Livsutvikling og livsanskuelse [Life’s development and perspectives on life]’, *Kirke og Kultur*, 1929.

11 For example, JR. Kyllingstad, *Measuring the Master Race: Physical Anthropology in Norway 1890–1945* (Cambridge, UK: Open Book Publishers, 2014); JR. Kyllingstad, ‘Norwegian Physical Anthropology and the Idea of a Nordic Master Race’, *Current Anthropology*, 2012, 53, 5, 46–56; 10:4. 1–12. A. Schanche, ‘Saami Skulls and Race Research in Norway’ in Fforde C, Turnbull P, Hubert J, eds, *The Dead and Their Possessions: Repatriation in Principle, Policy and Practice* (London: Routledge, 2002), 47–58; Evjen, ‘Measuring heads’.

12 A. Keskitalo, ‘Sametinget og samiske skjeletter i de Schreinerske samlinger [The Sami Parliament and Sami skeletons in the Schreiner Collection]’ *Sametinget [The Sapmi Parliament]*. 26 June 2020. https://sametinget.no/aktuelt/kronikk-sametinget-og-samiske-skjeletter-i-de-schreinerske-samlinger.7387.aspx; J. R. Måsø and A. B. Verstad, ‘Hodeskallejergerne [The Skull Hunters]’ *NRK*. 20 June 2020. https://www.nrk.no/sapmi/xl/raseforskere-jaktet-pa-hodeskaller-_-na-vil-ingar-ha-hjem-tippoldemoren-1.15031837#intro-authors--expand; L. Wade, ‘A racist scientist built a collection of human skulls. Should we still study them?’ *Science Magazine.* 8 July 2021; A. Svestad, ‘What happened in Neiden? On the Question of Reburial Ethics’ *Norwegian Archeological Review*, 2013, 46, 2, 194–222.

13 F. Bethencourt, *Racisms: From the Crusades to the Twentieth Century* (Princeton: Princeton University Press, 2013); S. J. Gould, *The Mismeasure of Man* (Harmondsworth: Penguin, 1992); I. Hannaford, *Race – The History of an Idea in the West* (Washington, DC: The Woodrow Wilson Center Press and Baltimore: The Johns Hopkins University Press, 1996); Ian Hacking, ‘Why Race Still Matters’, *Daedalus (Cambridge, Mass.)* 2005, 134, 1, 102–16; Cornel West, ‘A Genealogy of Modern Racism’, in P. Essed and D. T. Goldberg, eds, *Race Critical Theories* (Oxford: Blackwell, 2002), 90–112.

14 Hacking, ‘Why Race Still Matters’, 104.

15 Andreas Winkelman, ‘Repatriations of human remains from Germany – 1911 to 2019’, *Museum & Society*, 2020, 18, 1, 40–51.

16 Ann Fabian, *The Skull Collectors* (Chicago: University of Chicago Press, 2010); David Hunt, ‘History and Collections of the Division of Physical Anthropology, National Museum of Natural History, Smithsonian Institution’ in M. W. Warren, H. A. Walsh-Haney and L. Freas, eds, *The Forensic Anthropology Laboratory* (Boca Raton: CRC Press, 2008), 149–80; Christine Quigley, *Skulls and Skeletons: Human Bone Collections and Accumulations* (Jefferson, NC: McFarland, 2001); Samuel J. Redman, *Bone Rooms: From Scientific Racism to Human Prehistory in Museums* (Cambridge, MA: Harvard University Press, 2016).

17 Andrew Gulliford, ‘Bones of Contention: The Repatriation of Native American Human Remains’, *The Public Historian,* 1996, 18, 4, 120.

18 R. Thornton, ‘Repatriation as healing the wounds of the trauma of history: the cases of Native Americans in the United States of America’ in Fforde C., Turnbull P., Hubert J., eds, *The Dead and Their Possessions: Repatriation in Principle, Policy and Practice* (London: Routledge, 2002), 17–24; A. Lill, ‘After 87 Years At The Smithsonian, Bones Of Alaska Natives Returned And Reburied’, *NPR*. 21 October 2017. (https://www.npr.org/2017/10/21/554598592/after-87-years-at-the-smithsonian-bones-of-alaska-natives-returned-and-reburied)

19 L. T. Smith, *Decolonizing Methodologies: Research and Indigenous Peoples* (London: Zed Books, 1999); C. Fforde, ‘Vermillion Accord on Human Remains (1989) (Indigenous Archaeology)’, in Smith, C. ed, *Encyclopedia of Global Archaeology* (New York: Springer, 2014), 7612–15; P. Turnbull, ‘Vermillion Accord on Human Remains (1989) (Legislation)’, in Smith, C. (eds) *Encyclopedia of Global Archaeology* (New York: Springer, 2014), 7615–17; Gulliford, ‘Bones of Contention’; Quigley, ‘Skulls and Skeletons’; Redman, ‘Bone Rooms’; Fabian, ‘The Skull Collectors’.

20 Stephen Nash and Chip Colwell, ‘NAGPRA at 30: The Effects of Repatriation’, *Annual Review of Anthropology*, 2020, 49, 1, 225–39.

21 Ibid. 226.

22 Jane Buikstra, ‘Reburial: How We All Loose?’, *Council for Museum Anthropology Newsletter*, 1983, 7, 2, 2–5; J. E. Buikstra and C. C. Gordon, ‘The Study and Restudy of Human Skeletal Series: The Importance of Long-Term Curation’, *Annals of New York Academy of* Sciences, 1981, 376, 1, 449–65; D. Ubelaker and L. G. Grant, ‘Human Skeletal Remains: Preservation or Reburial?’, *Yearbook of Physical Anthropology*, 1989, 32, 249–87; Quigley, ‘Skulls and Skeletons’; Hunt, ‘History and Collections of the Division of Physical Anthropology’.

23 Hunt, ‘History and Collections of the Division of Physical Anthropology’, 173.

24 Ubelaker and Grant, ‘Human Skeletal Remains: Preservation or Reburial?’, 250.

25 Nash and Colwell, ‘NAGPRA at 30: The Effects of Repatriation’.

26 Hallvard Fossheim, ‘Research on Human Remains: An Ethics of Representativeness’, in K. Squires, D. Errickson and N. Márquez-Grant, eds, *Ethical Approaches to Human Remains: A Global Challenge in Bioarchaeology and Forensic Anthropology* (Cham, Switzerland: Springer, 2019); Per Holck, Den fysiske antropologi I Norge – Fra anatomisk institutts historie 1815–1990 [Physical Anthropology in Norway – From the History of the Institute of Anatomy 1815–1990], *vol*. 3. Antropologiske Skrifter (Oslo: University of Oslo, 1990); Berit Sellevold, ‘Norway’, in N. Márquez-Grant and Linda Fibiger, eds, *The Routledge Handbook of Archaeological Human Remains and Legislation: An International Guide to Laws and Practice in the Excavation and Treatment of Archaeological Human Remains* (New York: Routledge, 2010); A. Svestad, ‘What happened in Neiden? On the Question of Reburial Ethics’. *Norwegian Archeological Review*, 2013, 46, 2, 194–222. A. Schanche, ‘Knoklenes Verdi: Om forskning på og forvaltning av skjelettmateriale fra samiske graver’ [The Value of the Bones: On Research on and Management of Human Remains From Sami Graves], in Den Nasjonale Forskningetiske Komitee for Samfunnsvitenskap og Humaniora (NESH), *Samisk Forskning og Forskningsetikk* [Sami Research and Ethics] Publikasjon 2002, 2, 99–133.

27 T. Hamran, ‘Bring hodeskallene hjem’ [Bring the skulls home]. *NRK* 12 November 2007 (https://www.nrk.no/tromsogfinnmark/--bring-hodeskallene-hjem-1.3987415); E. Lieungh, ‘Samiske skjelett returneres’ [Sami skeletons are to be returned]. *NRK* 30 March 2011 (https://www.nrk.no/tromsogfinnmark/samiske-skjelett-returneres-1.7573034).

28 Svestad, ‘What Happened in Neiden?’

29 Svestad, ‘What Happened in Neiden?’, 202.

30 Quoted from a meeting in Neiden in 2007, in Svestad, ‘What Happened in Neiden?’, 194.

31 Venke Tørmænen, quoted in Svestad, ‘What Happened in Neiden?’, 201.

32 Quoted in Svestad, ‘What Happened in Neiden?’, 201.

33 The category of race was first introduced by the French physician Francois Bernier in 1684, and he divided humankind into three principal racial types: the Negroid, the Mongoloid and the European. In addition came the Sami (or Lapps) that he decided had to be regarded as a fourth type. See Evjen, ‘Measuring heads’, 4; West, ‘A Genealogy of Modern Racism’, 99.

34 Paul Brown, ‘Race and Racism’, in J. H. Birx, ed, *21st Century Anthropology* (Thousand Oaks: SAGE Publications, 2010), 65–75; Staffan Müller-Wille, ‘Linnaeus and the Four Corners of the World’, in A. C. Kimberly, R. Bauer, Z. Nunes and C. L. Peterson, eds, *The Cultural Politics of Blood, 1500–1900* (Basingstoke: Palgrave MacMillan, 2015). 191–209.

35 Hannaford, ‘Race-The history of an idea in the West’, 260.

36 Evjen, ‘Measuring Heads’, 7. The French neuroanatomist and anthropologist Paul Broca (1824–1880) expanded the index to consist of three main types, upgrading the mesocephaler from simply being an intermediary type. See John Haller, ‘Concepts of Race Inferiority in Nineteenth-Century Anthropology’ *Journal of the History of Medicine and Allied Sciences*, 1970, 25, 1, 40–51.

37 Nils Roll-Hansen, ‘Commentary: Wilhelm Johannsen and the problem of heredity at the turn of the 19th century’, *International Journal of Epidemiology*, 2014, 43, 4, 1007–13.

38 I. H. Stamhuis and A. Monsen, ‘Kristine Bonnevie, Tine Tammes and Elisabeth Schiemann in Early Genetics: Emerging Chances for a University Career for Women’ *Journal of the History of Biology*, 2007, 40, 3, 427–66; J. R. Kyllingstad, ‘Kristine Bonnevies vitenskapelighet og rasehygienen [Kristine Bonnevie, science and racial hygiene]’, *Arr*, 2019, 1–2; Kyllingstad, ‘Measuring the Master Race’; Roll-Hansen, ‘Norwegian Eugenics’.

39 University of Copenhagen, ‘The archaeo-anthropological collection’, 2021, https://retsmedicin.ku.dk/english/research-in-forensic-medicine/denantropologiskesamling_uk/

40 University of Uppsala, ‘The Historical Collections’, 2021, https://www.uu.se/en/gustavianum/our-collections/the-historical-collections/the-anatomical-collection/

41 University of Lund, ‘Lund University returns remains to Australia’, 2018, https://www.lunduniversity.lu.se/article/lund-university-returns-remains-australia

42 Karolinska Institutet, ‘FAQ: human remains in historical collections at KI’, 2021, https://ki.se/en/about/faq-human-remains-in-historical-collections-at-ki

43 University of Oslo, ‘The Schreiner Collection’, 2021, https://www.med.uio.no/imb/english/research/about/schreiner-collection/

44 Schanche, ‘Knoklenes verdi’, 102. B. J. Sellevold, ‘Skeletal Remains of the Norwegian Saami’ in Fforde C., Turnbull P., and Hubert J., eds, *The Dead and Their Possessions: Repatriation in Principle, Policy and Practice* (London: Routledge, 2002), 59–62.

45 Svestad, ‘What Happened in Neiden’.

46 Kyllingstad, ‘Measuring the Master Race’.

47 A Schreiner, *Slegtslivet hos menneskene*.

48 Kyllingstad, ‘Measuring the Master Race’, 174.

49 Ibid, 137 and 144; Evjen, ‘Measuring Heads’, 13.

50 Audhild Schanche, *Graver i ur og berg – Samisk gravskikk og religion fra forhistorisk til nyere tid* [Graves in Rocks and Mountains – Sami Burial Costums and Religion from Prehistory to Modern Times] (Karasjok: Davvi Girji OS, 2000); J. R. Kyllingstad, ‘Norwegian Physical Anthropology’; Kyllingstad, ‘Measuring the Master Race’, 143; Evjen, ‘Measuring Heads’. Svestad, ‘What Happened in Neiden’.

51 Svestad, ‘What Happened in Neiden’; Schanche, ‘Knoklenes verdi’.

52 Kyllingstad, ‘Measuring the Master Race’, xvii; Evjen, ‘Measuring Heads’; Quigley, ‘Skulls and Skeletons’.

53 Megan J. Highet, ‘Body Snatching & Grave Robbing: Bodies for Science’, *History and Anthropology*, 2005, 16, 4, 438.

54 Kyllingstad, ‘Measuring the Master Race’, 139; Evjen, ‘Measuring Heads’, 4; A. Schanche, ‘Saami Skulls’.

55 Poul Duedahl, ‘Dansk Raceantropologi I Grønland [Danish anthropology of race in Greenland]’, *Historisk Tidsskrift*, 2003, 103, 2, 335–8; Schanche, ‘Graver I ur og Berg’, 49; Evjen, ‘Measuring Heads’, 14.

56 Evjen, ‘Measuring Heads’, 13.

57 Evjen, ‘Measuring Heads’; Kyllingstad, ‘Measuring the Master Race’; Schanche, ‘Graver I ur og berg’.

58 Kyllingstad, ‘Measuring the Master Race’, 73; Kyllingstad, ‘Norwegian Physical Anthropology’, 49; Evjen, ‘Measuring Heads’, 25.

59 Kyllingstad, ‘Measuring the Master Race’, 84.

60 Proctor, ‘From Anthropologie to Rassenkunde’, 141.

61 Martin quoted in Proctor, ‘From Anthropologie to Rassenkunde’, 141.

62 Martin quoted in Proctor, ‘From Anthropologie to Rassenkunde’, 142.

63 Proctor, ‘From Anthropologie to Rassenkunde’, 142.

64 Cf. note 62. According to Evjen, ‘Measuring Heads’, 13: ‘that it was not possible, on the basis of this Sami material, to point to particular physical characteristics that differentiated them from others’.

65 Gould, ‘The Mismeasure of Man’, adopted from Duedahl, ‘Dansk raceantropologi I Grønland’, 339, note 9.

66 Bethencourt, ‘Racisms’; Brown, ‘Race and Racism’; Fabian, ‘The Skull Collectors’.

67 Keskitalo, ‘Sametinget og samiske skjeletter’; J. R. Måsø and A. B. Verstad, ‘Hodeskallejergerne’.

68 Ellingson, Ter. *The Myth of the Noble Savage* (Berkeley: University of California Press, 2001).

69 A. Schreiner quoted in Evjen, ‘Measuring Heads’, 16. See also Kyllingstad, ‘Norwegian Physical Anthropology’, 54.

70 K. E. Schreiner quoted in Nielssen, ‘Visualising Invisibility’, 7. See also Schanche, ‘Saami Skulls’, 51.

71 Duedahl, ‘Dansk Raceantropologi i Grønland’, 342 (our translation).

72 Karl Löwith, *Meaning in History* (Chicago: Phoenix Books, University of Chicago Press, 1949).

73 Michel de Montaigne, ‘Of Cannibals’, in D. M. Frame (trans), *The Complete Essays of Montaigne* (Stanford, CA: Stanford University Press), 150–8.

74 Jean-Jacques Rousseau, *A Discourse on Inequality*, Maurice Cranston (trans) (London: Penguin, 1984). See also Ellingson, ‘The Myth of the Noble Savage’.

75 Young, Robert M. *Darwin’s Metaphor: Nature’s Place in Victorian Culture*. Cambridge: Cambridge University Press, 1985; Kyllingstad, ‘Measuring the Master Race’.

76 Helge Jordheim, ‘Against Periodization: Koselleck’s Theory of Multiple Temporalities’, *History and Theory*, 2012, 51, 2, 151–71.

77 Ibid.

78 Reinhardt Koselleck, *Futures Past: On the Semantics of Historical Time* (Cambridge, MA: MIT Press, 1985).

79 Koch, ‘Past Futures’, 332–3.

80 Kyllingstad, ‘Measuring the Master Race’, 29.

81 Evjen, ‘Measuring Heads’, 25; Hannaford, ‘Race’, 232–33.

82 Kyllingstad, ‘Measuring the Master Race’.

83 Evjen, ‘Measuring Heads’, 16.

84 Schanche, ‘Saami Skulls’, 51.

85 Poul Duedahl, ‘Når begreber dræber: en begrebshistorisk analyse af racebegrebet [When Concepts Kill: A Conceptual Analysis of Race]’, in P. H. Hansen and J. Nevers, eds, *Historiefagets teoretiske udfordring* [Theoretical Challenges in Historical Research], (Syddansk Universitetsforlag, 2004), 116; Kyllingstad, ‘Measuring the Master Race’, 106–7; Roll-Hansen, ‘Commentary’; Roll-Hansen, ‘Norwegian Eugenics’, 167.

86 Poul Duedahl, ‘Læst og påskrevet – Om racebegrepet hos Darwin [Truth be Told – on the Concept of Race in Darwin]’ *Slagmark*, 2009, 54, 37 (our translation).

87 See note 58.

88 K. E. Schreiner, ‘Europas menneskeraser’, 138 (our translation).

89 Ibid. K. E. Schreiner, ‘Europas menneskeraser’.

90 Ibid, K. E. Schreiner, ‘Europas menneskeraser’, 145 (our translation).

91 For a similar interpretation, see Per Haave, ‘Sterilisering av tatere 1934–1977 – en historisk undersøkelse av lov og praksis [Sterilisation of Romani 1934–1977 – A Historical Investigation of Law and Practice]’, (Oslo: Norges Forskninsråd [The Norwegian Research Council]), 2000.

92 Johannes Sløk, *Hva er idehistorie?* [What is Intellectual History?] (Oslo: Cappelen, 1970).

93 See Kühl, Stefan. *For the Betterment of the Race: The Rise and Fall of the International Movement for Eugenics and Racial Hygiene* (New York: Palgrave Macmillan, 2013).

94 Galton quoted in Koch, ‘Past Futures’, 333.

95 Kevles, ‘In the Name of eugenics’.

96 Koch, ‘Past Futures’, 338; Kyllingstad, ‘Measuring the Master Race’, 99–100; Roll-Hansen, ‘Norwegian Eugenics’, 156.

97 Roll-Hansen, ‘Norwegian Eugenics’, 160.

98 Koch, ‘Past Futures’, 334.

99 A. Schreiner, ‘Slegtslivet hos menneskene’, 172.

100 Ibid. 157, 159.

101 Ibid. 166.

102 Ibid. 170.

103 K. E. Schreiner, ‘Et ord om videnskap og dilettantisme’ (our translation).

104 Kyllingstad, ‘Measuring the Master Race’.

105 Nils Roll‐Hansen, ‘The Holist Tradition in Twentieth Century Genetics. Wilhelm Johannsen’s Genotype Concept’, *The Journal of Physiology*, 2014, 592, 11, 2431–8; Roll-Hansen, ‘Commentary’.

106 Nils Roll-Hansen, ‘Eugenics Before World War 2: The Case of Norway’, *History and Philosophy of the Life Sciences*, 1980, 2, 2, 269–98; Roll-Hansen, ‘Norwegian Eugenics’; Koch, ‘Past Futures’; Kyllingstad, ‘Measuring the Master Race’. For the more international picture, see Kühl, ‘For the Betterment’, 71–76.

107 Nils Roll-Hansen, ‘Geneticists and the Eugenics Movement in Scandinavia’, *The British Journal for the History of Science*, 1989, 22, 3, 338.

108 Kyllingstad, ‘Measuring the Master Race’; Roll-Hansen, ‘Norwegian Eugenics’, 155.

109 K. E. Schreiner, ‘Et ord om vitenskap og dilettantisme’; Kyllingstad, ‘Measuring the Master Race’; Kyllingstad, ‘Norwegian Physical Anthropology’.

110 Kyllingstad, ‘Norwegian Physical Anthropology’, 52.

111 J. R. Kyllingstad, ‘The Norwegian Association for Heredity Research and the Organized International Eugenics Movement. Expertise, Authority, Transnational Networks and International Organization in Norwegian Genetics and Eugenics (1919–1934)’. *Perspectives on Science* 2022, 30, 1, 81; Kyllingstad, ‘Measuring the Master Race’, 98–99; Roll-Hansen, ‘Norwegian Eugenics’, 158.

112 Kyllingstad, ‘The Norwegian Association’, 83, 93; Kyllingstad, ‘Measuring the Master Race’, 204; Kyllingstad, ‘Norwegian Physical Anthropology’, 52–53.

113 K. E. Schreiner, ‘Europas menneskeraser’, 143–4 (our translation).

114 Roll-Hansen, ‘The Holist Tradition in Twentieth Century Genetics’.

115 Kyllingstad, ‘Norwegian Physical Anthropology’, 54.

116 Gould, ‘Mismeasure of Man’.

117 A. Schreiner, ‘Livsutvikling og livsanskuelse’; Kyllingstad, ‘Measuring the Master Race’, 185.

118 A. Schreiner, ‘Livsutvikling og livsankuelse’, 461, quoted from Kyllingstad’s translation, ‘Measuring the Master Race’, 186.

119 Sebastian Normandin and Charles Wolfe, ‘Vitalism and the Scientific Image in Post-enlightenment Life Science, 1800–2010’ in S. Normandin and C. Wolfe, eds, *History, Philosophy and Theory of the Life Sciences, vol. 2*. (Dordrecht: Springer, 2013).

120 P. W. Zappfe, ‘The Last Messiah’, *Philosophy Now*, 2004, 45. (https://philosophynow.org/issues/45/The_Last_Messiah).

121 A. Schreiner, ‘Livsutvikling og livsanskuelse’, 460 (our translation).

122 Ibid. 459.

123 S. J. Gould and R. C. Lewontin, ‘The Spandrels of San Marco and the Panglossian Paradigm: A Critique of the Adaptationist Programme’ *Proceedings of the Royal Society of London. Series B, Biological Sciences*, 1979, 205, 1161, 585.

124 A. Schreiner, ‘Livsutvikling og livsanskuelse’, 469.

125 Law on access to sterilisation of 1 June 1934, appendix to Per Haave, ‘Sterilisering av tatere’.

126 Roll-Hansen, ‘Norwegian Eugenics’, 169–76; Kyllingstad, ‘The Norwegian Association’, 102.

127 Noah Berlatsky, ‘The “Product of Its Time” Defense: No Excuse for Sexism and Racism’, *The Atlantic*, 28 January 2014. https://www.theatlantic.com/entertainment/archive/2014/01/the-product-of-its-time-defense-no-excuse-for-sexism-and-racism/283352/

128 Staffan Müller-Wille, ‘Race and History: Comments From an Epistemological Point of View’, *Science, Technology, & Human Values*, 2014, 39, 4, 597–606.

129 Ibid.

130 Eivind Engebretsen, ‘Antoine Godeau et la pauvreté exemplaire’, *Dix-septieme siecle*, 2011, 2, 351–70.

## Notes

Clemet Askheim has a MA in Philosophy and is presently a PhD fellow and affiliate researcher at the Centre for Sustainable Healthcare Education, Faculty of Medicine, University of Oslo.

Eivind Engebretsen has a PhD in Intellectual History and is currently professor of medical epistemology and Director of the Sustainable Health Unit (SUSTAINIT) at the Faculty of Medicine, University of Oslo.

Ivar Prydz Gladhaug holds an MD and a PhD in Cancer Biology. He is a professor of surgery at the University of Oslo, consultant in cancer surgery at Oslo University Hospital and the former Dean of the Faculty of Medicine, University of Oslo.

